# Prostate Radiotherapy in the Era of Advanced Imaging and Precision Medicine

**DOI:** 10.1155/2016/4897515

**Published:** 2016-02-16

**Authors:** Caleb R. Dulaney, Daniel O. Osula, Eddy S. Yang, Soroush Rais-Bahrami

**Affiliations:** ^1^Department of Radiation Oncology, University of Alabama at Birmingham, Birmingham, AL 35249-6832, USA; ^2^Department of Urology, University of Alabama at Birmingham, Birmingham, AL 35294, USA; ^3^Department of Radiology, University of Alabama at Birmingham, Birmingham, AL 35294, USA

## Abstract

Tremendous technological advancements in prostate radiotherapy have decreased treatment toxicity and improved clinical outcomes for men with prostate cancer. While these advances have allowed for significant treatment volume reduction and whole-organ dose escalation, further improvement in prostate radiotherapy has been limited by classic techniques for diagnosis and risk stratification. Developments in prostate imaging, image-guided targeted biopsy, next-generation gene expression profiling, and targeted molecular therapies now provide information to stratify patients and select treatments based on tumor biology. Image-guided targeted biopsy improves detection of clinically significant cases of prostate cancer and provides important information about the biological behavior of intraprostatic lesions which can further guide treatment decisions. We review the evolution of prostate magnetic resonance imaging (MRI) and MRI-ultrasound fusion-guided prostate biopsy. Recent advancements in radiation therapy including dose escalation, moderate and extreme hypofractionation, partial prostate radiation therapy, and finally dose escalation by simultaneous integrated boost are discussed. We also review next-generation sequencing and discuss developments in targeted molecular therapies. Last, we review ongoing clinical trials and future treatment paradigms that integrate targeted biopsy, molecular profiling and therapy, and prostate radiotherapy.

## 1. Introduction

Prostate cancer is the most common solid organ malignancy in American men with an estimated 220,800 newly diagnosed cases and projected 27,540 deaths for the year 2015 [1]. Prostate cancer screening was originally performed via the digital rectal exam (DRE). While still routinely performed and an important factor in risk stratification, the DRE is a limited screening tool as it bears subjectivity and primarily detects larger palpable lesions in the posterior prostate through the rectal vault. In fact, studies examining the utility of DRE in prostate cancer screening fail to demonstrate a reduction in cancer specific mortality in any age group [2]. In the 1980s, prostate specific antigen (PSA) and the transrectal ultrasound (TRUS) revolutionized the screening process for prostate cancer. Using PSA as a screening tool, the incidence of prostate cancer more than doubled from the 1970s to the 1990s. Ever-changing absolute PSA thresholds, age adjusted PSA thresholds, and PSA dynamic parameters have been used to trigger TRUS-guided biopsy.

The current method of using DRE, PSA, and TRUS biopsy to determine treatment has come under scrutiny. While the incidence of prostate cancer has risen with this screening algorithm, cases of clinically significant disease still go unrecognized and there is concern for overtreatment of more indolent, clinically insignificant cancers as current methods are not able to effectively detect patients who would render a survival benefit from definitive treatment [3, 4]. Furthermore, this screening process carries significant risk of infectious complications with antibiotic resistant organisms as well as downstream costs of treatment and treatment-related side effects and complications [5]. These problems have prompted search for alternative, more effective methods of screening for clinically significant prostate cancer.

## 2. Advances in Prostate Cancer Detection and Biopsy

### 2.1. Evolution of Multiparametric MRI in Prostate Cancer Detection

In the 1990s, clinicians began using magnetic resonance imaging (MRI) as a tool for staging men diagnosed with prostate cancer. The primary utilization of MRI at that time was identification of extracapsular extension and seminal vesicle invasion because early techniques poorly visualized intraprostatic lesions [5, 6]. The addition of an endorectal coil improved the signal-to-noise ratio of prostate MRI allowing for higher resolution T2-weighted (T2W) imaging and enhanced delineation of the prostatic capsule. Improved technology made MRI increasingly useful in identifying and characterizing lesions within the prostate as well as detecting local disease recurrence following primary definitive treatment [7, 8]. An early apparent advantage of MRI was preferential detection of high-risk features in large or more aggressive tumors compared to low grade tumors.

On T2W MRI, hypointense intraprostatic lesions correlate well with cancerous foci found in radical prostatectomy specimens. Similarly, these tumor foci also tend to preferentially enhance dynamic contrast enhanced (DCE) MRI series. The development of magnetic resonance spectroscopic imaging (MRSI), a functional study that detects relative levels of choline and citrate within tumors, adds to the specificity of MRI for intraprostatic lesions [8]. Diffusion-weighted imaging (DWI) is also useful in detecting prostate cancer. Quantitative evaluation of DWI with calculated apparent diffusion coefficient (ADC) values correlates with Gleason grade, making it applicable in risk stratification [9]. Combining MRI modalities, including T2W, DCE, and DWI, improves visualization and accurate detection of intraprostatic lesions. Furthermore, MRI improves the ability to detect central and anterior prostate cancers that are not routinely sampled on standard TRUS biopsies [10, 11].

The inclusion of multiple MRI parameters is known as multiparametric MRI (mpMRI). Overlapping modalities in the mpMRI approach corrects for deficiencies inherent in any individual sequence. The use of 2 or more parameters improves the accuracy of detection and localization of prostate cancer [12–15]. Combining the functional characteristics of different modalities also differentiates between low and intermediate/high-grade disease [16–18]. Increased utilization of mpMRI to detect and diagnose prostate cancer could lead to a decrease in biopsy and treatment utilization of patients with clinically insignificant disease.

While mpMRI provides valuable anatomic information that often correlates with high-risk histopathology, tissue diagnosis is still essential and remains the gold standard for diagnosing prostate cancer. Recent technological advances have allowed for the integration of mpMRI with ultrasound guided biopsies and this is currently being evaluated as a potential alternative or supplement to the standard TRUS biopsy. Three approaches have emerged that use mpMRI for guiding prostate biopsies including direct “in-bore” MRI biopsies, cognitive fusion, and MRI-TRUS fusion-guided biopsy [19].

### 2.2. MRI “In-Bore” Guided Biopsy

Initial studies using mpMRI to guide biopsy performed the biopsy under direct visualization in the MRI gantry. The patient first gets a diagnostic mpMRI and returns for biopsy if suspicious lesions are identified. Upon return, biopsies of the lesions are obtained under direct visualization using serial MRI scans to confirm biopsy needle placement. Advantages of this method are that only visualized lesions are biopsied, which decreases the total number of biopsies the patient receives, and this allows for precise documentation of biopsy needle locations [20–22]. The disadvantages of this technique are cost and patient tolerance. The closed magnetic environment requires the use of nonmagnetic needles and other supplies which are expensive and limit accessibility should a patient need immediate intervention. There have been a limited number of studies recording the utility of direct in-bore biopsies. One notable study performed by Hambrock et al. compared mpMRI with a 10-core TRUS and found that in-bore MRI-guided biopsies performed significantly better than TRUS-guided biopsies in predicting final pathology after radical prostatectomy (88 versus 55%,  *p* = 0.001) [23].

### 2.3. Cognitive Fusion Biopsy

Cognitive fusion biopsy is the simplest method of combining mpMRI and prostate biopsy. The urologist reviews previously acquired mpMRI images and then biopsies the general location of suspicious MRI lesions using the standard TRUS biopsy technique. The advantage of cognitive fusion biopsy is that it requires no additional equipment or cost, making it most easily adaptable to current practice models. The main disadvantage is strong operator dependency in correlating static MRI findings with dynamic real-time ultrasound findings. Cognitive fusion biopsy also lacks the ability to archive the exact location of the biopsy which could be important for focal therapy or surveillance purposes. Despite these potential shortcomings, the use of cognitive fusion biopsies increases prostate cancer detection and more accurately depicts overall disease burden in high-grade disease [24, 25]. Specifically, one study demonstrated prostate cancer detection rates up to 10% higher (15% for high-grade disease) with cognitive fusion biopsy compared to systematic biopsies in a similar population of patients [26].

### 2.4. MRI-TRUS Fusion Biopsy

The newest and most promising form of MRI-targeted prostate biopsy is the fusion of mpMRI with real-time TRUS imaging with postimage processing and software technology. In MRI-TRUS fusion biopsy, a diagnostic mpMRI is used to localize the tumor and a specialized software program fuses these images to a real-time TRUS image seen in the biopsy suite. An important practical advantage of MRI-TRUS fusion biopsy is that the MRI and the TRUS do not have to be physically or temporally linked. MRI data is transferred to one of several models of fusion software enabled 3D-TRUS units that can be located in a standard ultrasound suite. After upload, images of the prostate are remodeled using identification of landmarks (e.g., points, curves, and surfaces) that are present on both the MRI and TRUS platform. Since the prostate on MRI (with or without an endorectal coil) often differs in shape and contour from the same image on TRUS, the superimposed image must be transformed before successful fusion can occur. This is done through either an elastic or rigid transformation or a combination of both fusion algorithms. These images are shown as either a side-by-side display of the MRI and TRUS images or a single fused image allowing for targeted biopsy of the predelineated regions of interest from the diagnostic mpMRI on the real-time TRUS after fusion. The fusion enabled 3D-TRUS contains a tracking method that fixes the prostate in a 3D coordinate system so that movements of the US probe are mirrored on the fused MRI display. While this method appears to be less operator dependent, there is still need for operator input to assess and adjust altered gland contours or misregistration artifacts.

The data supporting the use of MRI-TRUS fusion biopsy is promising. Puech et al. compared the effectiveness of standard 12-core biopsy and MRI-TRUS fusion biopsy and found that fusion biopsy detected 10% more prostate cancer overall and 15% more clinically significant prostate cancer [26]. In the diagnostically challenging patient population of men with negative standard biopsies and elevated PSA, fusion biopsy detects 40% more clinically significant cancers but just 15% of clinically insignificant cancers compared to repeat standard biopsy [27]. Siddiqui et al. compared standard sextant TRUS biopsy, fusion biopsy, and combined biopsies. Out of 1003 patients, MRI-TB diagnosed 461 cases of prostate cancer and standard biopsy diagnosed 469. Among these, fusion biopsy diagnosed 30% more high-risk cancers and 17% fewer low-risk cancers compared to standard biopsy. In the 170 patients who went on to receive prostatectomies, fusion biopsy was more accurate (73%) than standard (59%) or the 2 combined (69%) in diagnosing intermediate- to high-risk disease [28]. These results have been replicated in several studies and suggest that MRI-TRUS fusion biopsy is superior to standard TRUS biopsy in detecting clinically significant disease and excluding insignificant disease, and it will play a prominent role in the future of the prostate cancer diagnosis and surveillance [29–32].

## 3. Prostate Radiotherapy Advances

Radiation therapy has been a mainstay in the treatment of prostate cancer since the 1960s with the development of high-energy teletherapy units and linear accelerators. Shortly thereafter, interstitial prostate brachytherapy became a primary treatment modality for organ-confined prostate cancer. Major advances in diagnostic imaging since that time have dramatically improved the ability to accurately target the prostate with smaller and smaller treatment volumes. This, in turn, led to better toxicity profiles, safe dose escalation, and improved disease control [33–39]. More recently, on-board imaging devices used to image the prostate during treatment have led to further increase in dose delivered per treatment and an associated decrease in total treatment duration. Trends toward earlier diagnosis during the PSA screening era have led to detection of more focal and smaller volume disease within the prostate. In an effort to deintensify treatment and avoid adverse effects in these patients, focal ablative techniques have been used to target only intraprostatic lesions as opposed to traditional treatment of the whole gland. Furthermore, in the era of precision medicine, advances in our understanding of cancer biology have led to genomic tests that describe the biological behavior of tumors and their risks for adverse outcomes. These tests allow the clinician to personalize prostate cancer therapy when combined with existing techniques.

### 3.1. Radiation Dose Escalation

The evolution of external beam radiation techniques and advanced imaging techniques has allowed for increasingly focal radiation therapy with margins around the prostate as small as 5 mm when using standard fractionation schemes [33]. Significant reduction in margins around the prostate, and thus volume of irradiated normal tissue, has been made possible by the use of daily on-board (cone-beam computed tomography) imaging prior to each treatment delivery [34]. Historically, the prostate was treated with four static radiation fields targeting a generous pelvic volume based on anatomic landmarks. With advancements in imaging, more focal three-dimensional treatment plans were developed to target the prostate and seminal vesicles only. Further advances in radiation delivery techniques such as intensity modulated radiation therapy (IMRT) and volumetric modulated arc therapy (VMAT) led to greater sparing of adjacent normal tissue to reduce toxicity. Lastly, on-board imaging has allowed daily localization of the prostate and/or fiducial markers to further narrow target volume margins. Improved accuracy and organ avoidance thus provided the opportunity to investigate dose escalation as a means of improved disease control. Retrospective series at that time demonstrated both an apparent dose response relationship for prostate cancer with improved local control and no significant toxicity increase when dose was increased using conformal techniques. Five large randomized trials (Table 1) have demonstrated that increased dose to the prostate of 74–80 Gray (Gy) in standard 1.8–2 Gy fractions results in improved biochemical recurrence-free survival and disease specific survival [35–39]. A large population study has demonstrated improved overall survival with dose escalation in men with high- and intermediate-risk prostate cancer suggesting that, in large enough populations, improved biochemical control can translate into a survival benefit [40].

### 3.2. Proton Therapy

A second strategy toward dose escalation involves heavy ion-based irradiation such as proton therapy. Proton therapy differs from conventional photon-based radiation therapy in that protons are charged particles that deposit a higher proportion of energy toward the end of their path of travel in a tissue and little to no energy beyond. Therefore, a very steep dose gradient can be created to minimize dose spill into adjacent tissue as compared to photon therapy. Unfortunately, population studies and experiences from large proton centers do not show superiority in disease control or toxicity for proton therapy [41–43].

### 3.3. Hypofractionation

One of the disadvantages of dose-escalated fractionated radiation to the prostate is the prolonged duration of treatment using standard fractionation schemes of 1.8 to 2 Gy per fraction to total doses of 74 to 80 Gy. In addition, there is a biological rationale for delivering higher radiation dose over a shorter period of time. Thus, multiple phase III trials have been conducted to demonstrate the safety, feasibility, and efficacy of hypofractionated, or shorter than standard, regimens [44–53]. The biological rationale for hypofractionated radiation therapy is to take advantage of the hypothetical differences in radiation sensitivity between malignant and normal prostate, decrease time and cost of treatment, and further escalate dose with the intention of improved local control.

Cell survival after radiation therapy is modeled by an exponential function that accounts for both direct, called alpha, and indirect, called beta, mechanisms of DNA damage. The ratio, or alpha/beta ratio, of these types of damage can give a general sense of the ability of the tissue to repair that damage. This repair ability is inversely proportional to the alpha/beta ratio. Generally, normal tissues have an alpha/beta ratio around 3 and tumors around 10. Historically, most radiation treatment schedules have been designed to capitalize on these differences in damage repair between tumor and normal tissue by delivering small doses of radiation over a prolonged period of time. This is the case for standard fractionation prostate radiation. More recent data, however, suggests that prostate cancer may actually have a lower alpha/beta ratio than previously suspected. This would mean that there is less benefit to lower dose, fractionated regimens.

Using this rationale, recent studies have investigated shorter courses of radiation therapy with higher doses per treatment (Table 2). Two early, phase three hypofractionation trials were designed prior to the dose escalation era and demonstrated similar outcomes to non-dose-escalated standard fractionation therapy [44, 45]. Three later studies were designed to test the superiority of hypofractionated radiation for biochemical control compared to dose-escalated standard fractionation [47–49]. Outcomes in these trials were similar, including toxicity. Three more modern noninferiority trials have compared toxicity outcomes between standard and moderate hypofractionation regimens [46, 50–52]. RTOG 0415 recently reported the noninferiority of a 70 Gy at 2.5 Gy per fraction regimen and similar toxicity to standard fractionation [51]. In the other trials, toxicity has been similar with the exception of the HYPRO trial which shows worse early GI toxicity with hypofractionation [52]. The initial results of a fourth noninferiority trial (PROFIT) are pending at this time [53].

Extremely hypofractionated radiation regimens consisting of 5 treatments or less have also been investigated (Table 3). Three randomized trials are currently investigating the efficacy and toxicity of extreme hypofractionated regimens in comparison to standard fractionation (Table 3) [54–56]. In these trials, treatment consists of 5 to 7 fractions of 6.1 to 7.6 Gy per fraction. RTOG 0938 is a randomized phase II trial investigating two extreme hypofractionation regimens in patients with favorable risk prostate cancer [57]. Treatment is delivered over 2 to 2.5 weeks with either 36.25 Gy in 5 nonconsecutive fractions or 51.6 Gy in 12 daily fractions. Importantly, the short- and long-term toxicity profiles of these extreme hypofractionated regimens will need to be determined.

### 3.4. Focal Targeting of Intraprostatic Lesions

External beam radiation dose escalation and hypofractionation trials increased dose homogeneously to the entire prostate. Despite this, the most common location of recurrence is within the prostate [58]. 80% of prostate cancers, particularly higher grade cancers, have multiple foci of disease in the prostate gland as demonstrated in radical prostatectomy specimens.

More recent evidence supports the idea that dominant intraprostatic lesions, as opposed to multifocal disease, drive the natural course of disease. Furthermore, these dominant lesions are the site of most recurrences [59–61]. Unfortunately, it remains challenging to identify and target volumes within the prostate at the highest risk of harboring clinically relevant disease. In other disease sites, differential doses of radiation are delivered to different volumes depending on their perceived risk of tumor involvement. Prostate cancer has been classically diagnosed by needle biopsy sampling of the entire gland. TRUS is inaccurate in localizing focal disease. Transperineal template-guided prostate mapping biopsy (TTMP) has previously been the gold standard for localizing disease within the prostate, but this procedure is very invasive. Focal therapies, therefore, have primarily relied upon imaging, particularly MRI, to identify and target treatment to dominant lesions. Yet, there still remains the uncertainty between the appearance of a dominant lesion and its true biology with imaging alone. Now, with MRI-US fusion-guided biopsy, the imaging can be used to identify cancer presence and define grade in a targeted fashion with 3D mapping of the areas of interest as well as precise documentation of sites biopsied. Due to the concern regarding overtreatment of early stage disease and with technological improvements allowing more focal radiation delivery, many have sought to develop more focal therapies to avoid normal tissue toxicity related to definitive treatment.

Radiation techniques to deliver focal therapy to prostate lesions involve both external beam radiation and prostate brachytherapy. External beam techniques include IMRT, VMAT, and helical tomotherapy. Multiple dosimetric studies demonstrate the feasibility of escalating dose to an intraprostatic lesion up to 100 Gy with little to no potential for excess toxicity compared to standard whole-gland treatment. A single phase II trial has demonstrated feasibility of escalating dose to an intraprostatic lesion to 80 Gy with toxicity comparable to standard homogeneous dosing [62].

Unfortunately, sacrificing whole-organ dose for focal boost results in inferior biochemical control using both external beam and brachytherapy techniques, especially with intermediate and high-risk disease [63]. In the setting of whole-organ treatment, though, preliminary data from the ASCENDE-RT trial demonstrate improved RFS in men with intermediate- and high-risk disease who had brachytherapy boost over conventional external beam boost [64]. These data suggest that there is still a role for further dose escalation for high-risk prostate cancer. Similar benefit to HDR brachytherapy dose escalation after external beam radiation has also been demonstrated in randomized trials [65, 66].

**Figure 1 fig1:**
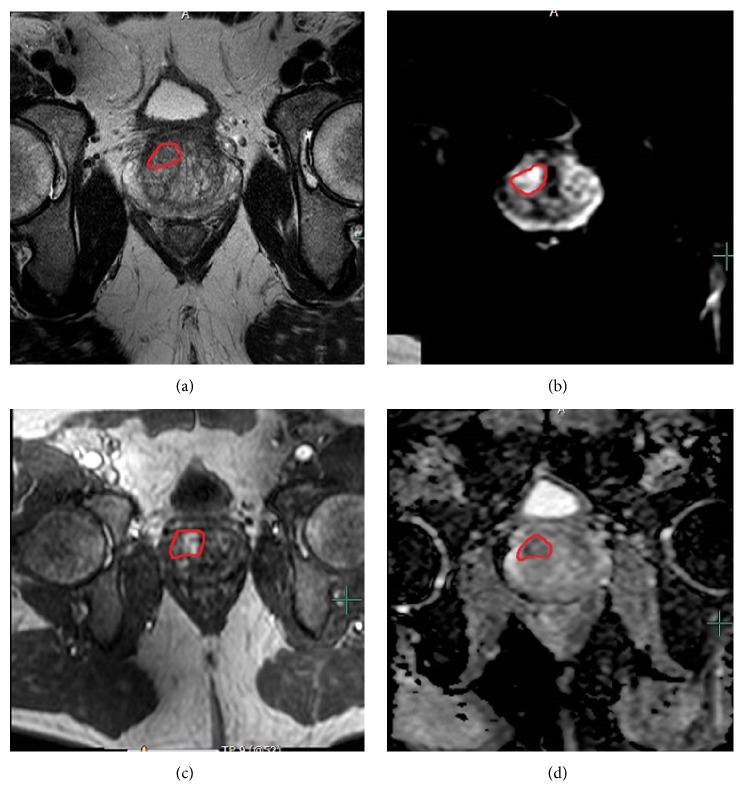
Multiparametric MRI evaluation and MRI-TRUS fusion biopsy in patient with multifocal intraprostatic lesions. The index lesions based upon MRI were identified in the right mid anterior central gland as an area of (a) T2 hypointensity, (b) increased signal on high *b*-value DW-MRI, (c) early enhancement on DCE-MRI, and (d) diffusion restriction on ADC map of DW-MRI. The right mid anterior central gland lesion demonstrated Gleason 3 + 4 disease on fusion biopsy. A second right base posterior peripheral zone lesion demonstrated Gleason 3 + 3 disease.

**Figure 2:**
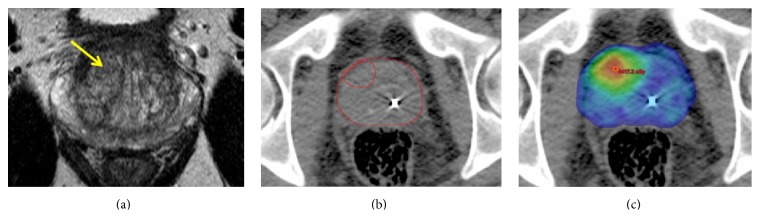
Axial views of the patient in Figure 1 with Gleason 3 + 4 disease found in right mid anterior central gland using MRI-TRUS fusion biopsy. The T2 hypointense lesion is shown in (a) with clinical target volumes drawn around the prostate and nodule on axial CT in (b). A 36 Gy dose colorwash to the whole prostate and simultaneous integrated boost of 40 Gy to the T2 hypointense lesion using an extreme hypofractionation radiation treatment plan are shown in (c). Note the fiducial markers used for daily image-guided localization.

The approach used in current prostate radiotherapy trials investigating hypofractionation and extreme hypofractionation utilizes a technique called simultaneous integrated boost (SIB) to deliver higher dose to dominant intraprostatic lesions while still delivering an adequate lower dose to the whole prostate. [Fig fig1] Figure 2 shows the dose distribution within the prostate of an extreme hypofractionation SIB plan. However, this technique continues to rely on radiographic assessment of risk of intraprostatic lesions and correlation with sextant biopsy to guide focal therapy.

### 3.5. Tumor Biology Directed Treatment Intensification

We now know that delivering higher radiation doses benefits men with the most high-risk disease. We also know that only targeting individual lesions within the prostate in these men leads to worse outcomes. Doses as high as 85 Gy have been delivered to the entire prostate with external beam radiation therapy [67]. The preliminary results of the ASCENDE-RT trial suggest that further dose escalation in high-risk disease has further potential benefit. However, increasing whole-prostate dose comes at the cost of increased toxicity. Therefore, the rational progression of these ideas leads to a treatment paradigm where the entire prostate is treated to an adequate dose with focal dose escalation to high-risk lesions within the prostate. This approach, in theory, could optimize dose escalation and normal organ toxicity. We may also find that this approach allows dose to be decreased to the whole-prostate and further escalated to intraprostatic lesions.

A newer approach to more accurately and appropriately intensify therapy is to combine information obtained by MR-TRUS fusion biopsy with SIB radiation therapy. Fusion biopsy identifies more clinically relevant, high-risk disease, which benefits most from treatment intensification. It also provides direct correlation between imaging and histopathologic findings. Furthermore, other intraprostatic lesions can be biologically risk-stratified to guide treatment planning. A phase II protocol at the University of Alabama at Birmingham (NCT01856855) is investigating the efficacy and toxicity of such an approach [68]. This protocol uses MR-TRUS fusion biopsy to guide selection of high-risk intraprostatic targets for escalated therapy. mpMRI is then used in the treatment planning process to identify the prostate and high-risk targets. The entire prostate is prescribed a total dose of 36.25 Gy in 5 fractions and high-risk volumes are prescribed a total dose of 40 Gy in 5 fractions using the SIB technique. Gold fiducial markers and daily cone-beam CT scans are used to accurately deliver the prescribed dose to the appropriate volume.

### 3.6. Genomic Predictors of Prostate Cancer Outcomes and Targeted Therapy

A big challenge in choosing adequate therapy and determining outcomes from clinical trials in prostate cancer is the significant disease heterogeneity within risk groups. Genomic and molecular analyses of prostate cancer specimens will hopefully help us better characterize disease risk and personalize treatment. Multiple genomic panels have been developed and validated in predicting outcomes based on tissue from radical prostatectomy or biopsy specimens. Prolaris (Myriad Genetics, Salt Lake City, UT, USA) is a 46-gene expression panel for biopsy and TURP specimens that predicts prostate cancer death. It has been validated in radical prostatectomy specimens to predict biochemical recurrence and distant metastases [69, 70]. Decipher (GenomeDX Biosciences, Vancouver, BC, Canada) is a 22-gene panel that predicts survival after radical prostatectomy [71]. Lastly, Oncotype DX Genomic Prostate Score (Genomic Health Inc., Redwood City, CA, USA) is a 17-gene panel that predicts recurrence, prostate cancer death, and high-risk pathology based on biopsy specimens. Genomic panels, applied to MR-TRUS biopsy samples, could potentially provide important information about the underlying biology of individual prostate lesions that could then be targeted with focally intensified therapy.

## 4. Summary

Despite significant advances in prostate cancer therapy over the last few decades, many men, particularly those with high-risk disease, will have PSA recurrence, develop symptomatic local or distant disease, or die from their prostate cancer. Prostate cancer is a heterogeneous disease and can even manifest heterogeneously within the prostate of a single patient. Prostate cancer therapy is moving rapidly toward personalization, and this approach could significantly improve outcomes for men with high-risk biology. Future clinical trials and standard therapy will biologically risk-stratify patients in order to optimize treatment outcome. Personalized prostate cancer therapy, therefore, will depend on our ability to accurately identify, biopsy, analyze, and treat areas of high-risk disease within the prostate for men with organ-confined disease. The incorporation of MR-TRUS biopsy, molecular testing of biopsy specimens, and focal treatment intensification will make personalized therapy a reality.

## Figures and Tables

**Table 1:** Randomized controlled trials evaluating the efficacy of radiation dose escalation for prostate cancer.

Trial	*N*, inclusion criteria	Dose comparison (Gray)	Outcome
MD Anderson [35]	301 cT1-3 N0 M0	70 versus 78	78% versus 59% Freedom from biochemical or clinical failure
PROG 95-09 [37]	393 cT1b-2b, PSA ≤ 15	70.2 versus 79.2	32% versus 17% 10-year biochemical failure
MRC RT01 [39]	843 cT1b-3a N0 M0, PSA < 50	64 versus 74	43% versus 55% 10-year biochemical recurrence-free survival
Dutch [36]	664 cT1b-4	68 versus 78	54% versus 64% Freedom from failure
GETUG 06 [38]	306 cT1b-3a N0 M0, PSA < 50	70 versus 80	39% versus 28% Biochemical failure

PSA: prostate specific antigen.

**Table 2:** Randomized controlled trials evaluating the efficacy and toxicity of hypofractionated radiation regimens.

	Trial	*N*, inclusion criteria	Dose (dose per fraction)	Outcome
Early hypofractionation trials	Lukka et al. [44]	936 GS 6–10	52.5 Gy (2.625 Gy) 66 Gy (2 Gy)	40% versus 43% 5-year freedom from biochemical failure
	Yeoh et al. [45]	217	55 Gy (2.75 Gy) 64 Gy (2 Gy)	53% versus 34% 7-year freedom from biochemical failure

Modern superiority trials	Hoffman et al. [47]	204 99% low-intermediate risk	72 Gy (2.4 Gy) 75.6 Gy (1.8 Gy)	96% versus 92% 5-year freedom from biochemical failure
	Pollack et al. [49]	303 GS 6+	70.2 Gy (2.7 Gy) 78 Gy (2.17 Gy)	23% versus 21% 5-year biochemical or clinical disease failure
	Arcangeli et al. [48]	168 GS 7+	62 Gy (3.1 Gy) 80 Gy (2 Gy)	85% versus 79% 5-year freedom from biochemical failure

Modern noninferiority trials	Dearnaley et al. [46]	457	57 Gy (3 Gy) 60 Gy (3 Gy) 74 Gy (2 Gy)	Similar GU and GI toxicity ≥ grade 2 (<5%)
	Incrocci [52]	820 Intermediate-high risk	64.6 Gy (3.4 Gy) 78 Gy (2 Gy)	Worse GI toxicity ≥ grade 2, similar GU toxicity
	RTOG 0415 [51]	1101 Low risk	70 Gy (2.5 Gy) 73.8 Gy (1.8 Gy)	Noninferior biochemical recurrence and overall survival, similar toxicity
	PROFIT [53]	Intermediate risk	60 Gy (3 Gy) 78 Gy (2 Gy)	Pending

GS: Gleason score. Gy: Gray.

**Table 3:** Randomized controlled trials evaluating the efficacy and toxicity of extreme hypofractionated radiation regimens.

Trial	Inclusion criteria	Dose (dose per fraction)
HYPO-RT-PC [54]	Intermediate risk	78 Gy (2 Gy) versus 42.7 Gy (6.1 Gy)
PACE [55]	Low-intermediate risk	(1) Radical prostatectomy versus 36.25 Gy (7.25 Gy) (2) 78 Gy (2 Gy) versus 36.25 Gy (7.25 Gy)
Proton cooperative group [56]	Low-intermediate risk	79.2 Gy (1.8 Gy) versus 38 Gy (7.6 Gy)
